# Nebivolol, a β1-adrenergic blocker, protects from peritoneal membrane damage induced during peritoneal dialysis

**DOI:** 10.18632/oncotarget.8780

**Published:** 2016-04-18

**Authors:** Georgios Liappas, Guadalupe González-Mateo, Anna Rita Aguirre, Hugo Abensur, Patricia Albar-Vizcaino, Emilio González Parra, Pilar Sandoval, Laura García Ramírez, Gloria del Peso, Juan Manuel Acedo, María A. Bajo, Rafael Selgas, José A. Sánchez Tomero, Manuel López-Cabrera, Abelardo Aguilera

**Affiliations:** ^1^ Immunology and Cellular Biology Department, Molecular Biology Centre Severo Ochoa, Madrid, Spain; ^2^ Nephrology Department, University of Sao Paulo, School of Medicine, Sao Paulo, Brazil; ^3^ Molecular Biology Unit and Nephrology Department, Hospital Universitario de La Princesa, Instituto de Investigación Sanitaria Princesa (IP), Madrid, Spain; ^4^ Nephrology Department, Fundación Jiménez-Díaz, Instituto de Investigación Sanitaria, Madrid, Spain; ^5^ Nephrology Department, Instituto de Investigación Hospital Universitario La Paz (IdiPAZ), Madrid, Spain; ^6^ Cardiology Department, Clínica La Luz, Madrid, Spain

**Keywords:** peritoneal dialysis, β-adrenergic receptor, nebivolol, type-I peritoneal membrane failure, peritoneal dialysis liquids, Pathology Section

## Abstract

Peritoneal dialysis (PD) is a form of renal replacement treatment, which employs the peritoneal membrane (PM) to eliminate toxins that cannot be removed by the kidney. The procedure itself, however, contributes to the loss of the PM ultrafiltration capacity (UFC), leading consequently to the technique malfunction. β-blockers have been considered deleterious for PM due to their association with loss of UFC and induction of fibrosis. Herein we analyzed the effects of Nebivolol, a new generation of β_1_-blocker, on PM alterations induced by PD fluids (PDF).

*In vitro:* We found that mesothelial cells (MCs) express β_1_-adrenergic receptor. MCs were treated with TGF-β to induce mesothelial-to-mesenchymal transition (MMT) and co-treated with Nebivolol. Nebivolol reversed the TGF-β effects, decreasing extracellular matrix synthesis, and improved the fibrinolytic capacity, decreasing plasminogen activator inhibitor-1 (PAI-1) and increasing tissue-type plasminogen activator (tPA) supernatant levels. Moreover, Nebivolol partially inhibited MMT and decreased vascular endothelial growth factor (VEGF) and IL-6 levels in supernatants.

*In vivo*: Twenty-one C57BL/6 mice were divided into 3 groups. Control group carried a catheter without PDF infusion. Study group received intraperitoneally PDF and oral Nebivolol during 30 days. PDF group received PDF alone. Nebivolol maintained the UFC and reduced PM thickness, MMT and angiogenesis promoted by PDF. It also improved the fibrinolytic capacity in PD effluents decreasing PAI-1 and IL-8 and increased tPA levels.

*Conclusion:* Nebivolol protects PM from PDF-induced damage, promoting anti-fibrotic, anti-angiogenic, anti-inflammatory and pro-fibrinolytic effects.

## INTRODUCTION

Peritoneal dialysis (PD) is a renal replacement therapy commonly used around the world. PD uses the peritoneal membrane (PM) as semipermeable barrier for water and solute exchange. Ultrafiltration failure (UFF) along with acute peritonitis are major causes of the technique dropout [1].

The factors associated to PM failure include chronic and acute peritoneal inflammation caused by bacteria, glucose degradation products, advanced glycation end-products, acidic pH from PD fluids (PDF), and hemoperitoneum [2, 3]. Activated inflammatory cells subsequently drive fibrotic and pro-angiogenic factors and stimulates mesothelial cells (MCs) to start trans-differentiation. This process is so-called mesothelial-to-mesenchymal transition (MMT), in which MCs acquire a fibroblast-like phenotype [4], lose their basoapical and basolateral polarity and acquire migratory capacity towards the submesothelium, where they synthesize extracellular matrix (ECM) and vascular endothelial growth factor (VEGF), thus contributing to PM structural damage. Not surprisingly, numerous efforts have been made to improve the PDF biocompatibility and to investigate drugs capable of neutralizing the PM damage [3].

The CMs monolayer is the first barrier exposed to different peritoneal aggressions. Its injury provokes aberrant expression of several genes, making these cells protagonists of peritoneal damage [5]

β-adrenergic receptors (β-AR) are a class of G protein-coupled receptors that are targets of catecholamines (noradrenaline and epinephrine). Many cells including pleural MCs [6] express these receptors, which participate in many biological functions and its blockade might be beneficial in fibrosis pathway [7].

β-AR blockers were introduced into the market more than four decades ago in order to control cardiac arrhythmias and blood pressure in cardiac and hypertensive patients. In PD patients their use was limited due to possible development of severe secondary effects, such as peritoneal sclerosis and UFF [8, 9]. One of the suggested reasons for UFF was the splanchnic arteriolar vasoconstriction especially associated with non-selective β-blockers administration. The consequence of splanchnic arteriolar vasoconstriction is the reduction in portal venous pressure and the PD ultrafiltration rate. In fact it is the rationale for prescribing β-AR blockers to cirrhosis patients with bleeding esophageal varices [10, 11]. Since then, new indications for β-blockers have been restored. Robust studies [12] have shown that patients suffering from heart failure and/or ischemic heart disease have a higher survival rate using β-blockers.

For these reasons and given that cardiovascular diseases are very frequent in renal replacement therapy patients [13], the use of b-blockers, especially those of new generation, must be reconsidered [14].

Nebivolol, is a highly selective β_1_-adrenergic receptor (β_1_-AR) blocker, whose mechanism of action is to increase nitric oxide (NO) release by endothelial cells, promoting a vasodilator effect and reducing the vascular resistance [14]. Nebivolol also decreased collagen synthesis, angiogenesis and shows an anti-oxidant effect [15] [16]. Recently it has been demonstrated that pleural MCs express β-AR [6], which participates not only in the tissue fibrosis inhibition but also in pleural fluid transport such as pleural lymphatic flow effusions [7]. Therefore, it is plausible that peritoneal MCs express β-ARs and that Nebivolol could protect the PM from the changes induced by PD (MMT, fibrosis, angiogenesis and UFF).

Herein, we demonstrate for the first time that human MCs express the β_1_-AR. Using a PD mice model [17], we observed that, though the blockade of this receptor with Nebivolol. Some deleterious effects associated with PD are reduced, such as MMT, PM fibrosis, angiogenesis and UFF, with an increase in the MCs fibrinolytic capacity.

## RESULTS

### Human omentum derived mesothelial cells (HOMCs) express b1-AR and its blockade with Nebivolol partially inhibits the MMT

**Figure 1:**
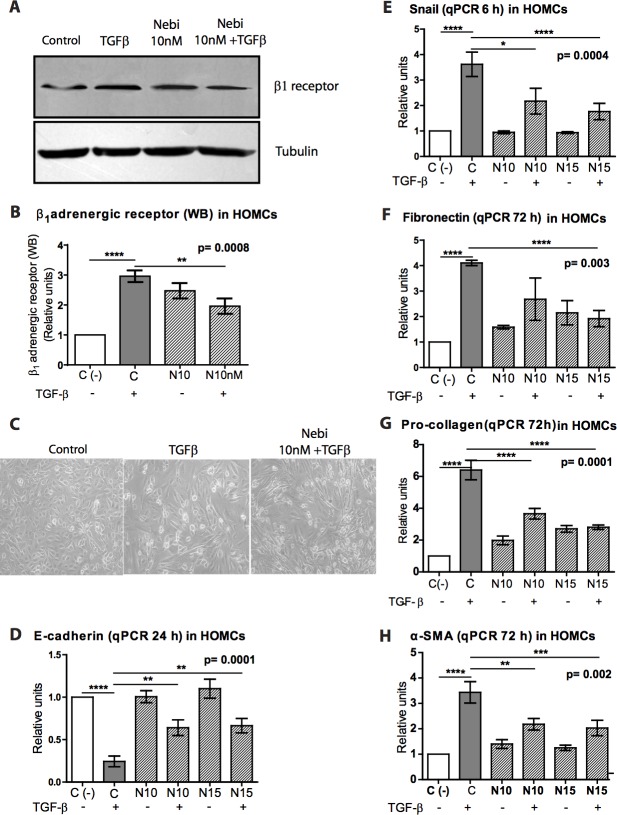
Effect of Nebivolol on b1-adrenergic receptor of HOMCs **A.** Immunoblotting analysis of β_1_-adrenergic receptor in reference to tubulin in protein lysates from omentum cells cultures treated, or not, with Nebivolol at 10 nM with or without TGF-β (1 ng/ml). **B.** The corresponding graph is shown. **C.** Representative pictures from cultures of omentum (control), TGF-β- and TGF-β + Nebivolol-treated cells **D.**-**H.** qPCR analyses of mRNA expressions of different MMT from omentum cell treated, or not, with Nebivolol at different concentrations (N10 = 10 nM or N15 = 15 nM) with or without TGF-β (1 ng/ml). (D) E-Cadherin (24 h), (E) Snail (6 h), (F) fibronectin (72 h), (G) pro-collagen (72 h) and (H) α-SMA (72 h). The markers were analyzed at different time points taking into consideration that the time of their high expression varies among them. Bars graphics represent means ± SE (*n* = 3).

Herein, we demonstrate for the first time that HOMCs show β1-AR (Figure 1A, 1B) and that Nebivolol (10 nM) decreased its expression. Recent publications show that β_1_-AR blockade is generally associated with anti-fibrotic and anti-angiogenic effects [18, 19]. Given that both effects are commonly associated to MMT and PM failure, we decided to explore the effect of Nebivolol on HOMCs stimulated with TGF-β. We measured the expression of genes that are usually altered during the MMT process (down-regulation of E-cadherin and up-regulation of Snail, fibronectin, pro-collagen and α-SMA, among others) [4].

The co-treatment with Nebivolol (10 nM) and TGF-β (1 ng/ml) did not inhibit the MMT phenotype (Figure 1C), however, this drug kept the E-cadherin expression at the level of 60% of its control value (Figure 1D). Likewise Nebivolol inhibited the up-regulation of Snail, fibronectin, pro-collagen and alpha smooth muscle actin (α-SMA), in a dose dependent manner (Figure 1E-1H).

### Nebivolol improved the fibrinolytic capacity and decreased the VEGF and IL-6 levels in HOMCs and human effluent derived mesothelial cells (HEMCs)

**Figure 2:**
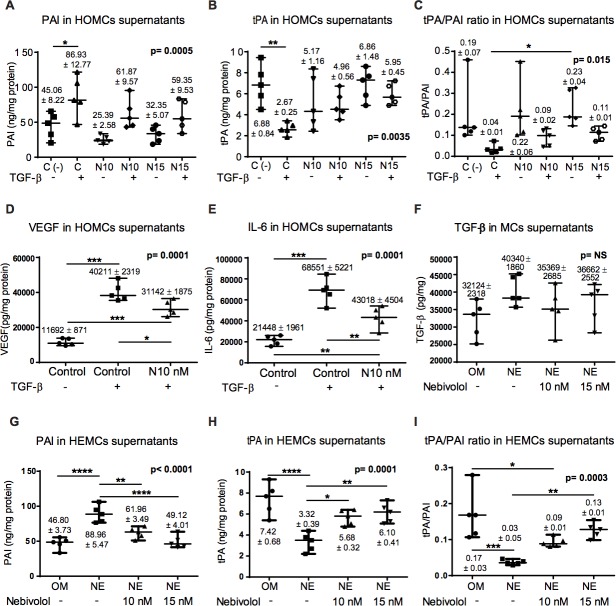
Effect of Nebivolol on fibrinolytic capacity, cytokines and growth factors in HOMCs and HEMCs **A**., **B**. Expression of the fibrinolytic factors PAI and tPA by HOMCs treated or not with TGF-β and with different doses of Nebivolol (10 or 15nM) during 48 h. **C**. The tPA/PAI ratio as fibrinolytic capacity marker was also determined. **D**. and **E**. VEGF and IL-6 supernatant levels in HOMCs treated with TGF-β. **F**. TGF-β levels in transdifferentiated HEMCs supernatant. **G.** and **H**. Expression of the fibrinolytic factors PAI and tPA by HEMCs treated with different doses of Nebivolol (10 or 15nM) during 48 h. **I**. Levels of tPA/PAI ratio in HEMCs. The levels of these factors were measured in HOMCs and HEMCs supernatants by ELISA and results are depicted as nanograms per milligrams of total cellular proteins. Data point graphics represent the absolute value of each determination and lines the median, lower and upper range. Numbers on the top of graphics represent the mean ± SE (*n* = 5). *P* values < 0.05 are considered statistically significant using one-way Anova test, and are depicted in the graphs. To account for multiple comparisons, the Bonferroni post-test was used to compare all pairs of means. The symbols represent the statistical differences between the groups (*p < 0.05, **p < 0.01, ***p < 0.001, ****p < 0.0001). NE = non epithelioid HEMCs; NS = no significant.

The fibrinolytic capacity of MCs is essential to maintain the production/degradation balance of extracellular matrix components in order to avoid the formation of peritoneal adhesions and fibrosis. Experimentally, the MMT induced by TGF-β_1_ was also associated to increase in plasminogen activator inhibitor-1 (PAI-1) level which is a strong anti-fibrinolytic molecule. Nebivolol tend to decrease PAI values in HOMCs supernatant, although this decrease does not reach statistical difference (Figure 2A). Moreover, the levels of its natural inhibitor, the tissue-type plasminogen activator (tPA), the most potent fibrinolytic factor known, were decreased upon TGF-β_1_ treatment. Nebivolol tended to restore the tPA baseline levels in HOMCs (Figure 2B) increasing tPA/PAI-ratio, a commonly used clinical marker for fibrinolytic capacity (Figure 2C).

Similar and even more powerful effects were observed in transdifferentiated HEMCs treated with Nebivolol. This pro-fibrinolytic effect also showed a dose-dependent pattern (Figure 2G to 2I).

TGF-β is a master molecule in both MMT induction and fibrosis pathogenesis. Therefore, we measured TGF-β levels in transdifferentiated HEMCs supernatant treated with Nebivolol. We did not find significant effect of Nebivolol on TGF-β supernatant levels (Figure 2F). VEGF and IL-6 are molecules related to angiogenesis and inflammation respectively. Treatment with Nebivolol decreased both VEGF and IL-6 supernatant levels in TGF-β-stimulated HOMCs (Figure 2D and 2E). These results suggest that Nebivolol improves the fibrinolytic capacity and might have an anti-angiogenic and anti-inflammatory effect.

### Nebivolol ameliorated peritoneal membrane alterations induced by dialysis fluid exposure in a PD model in mouse

**Figure 3:**
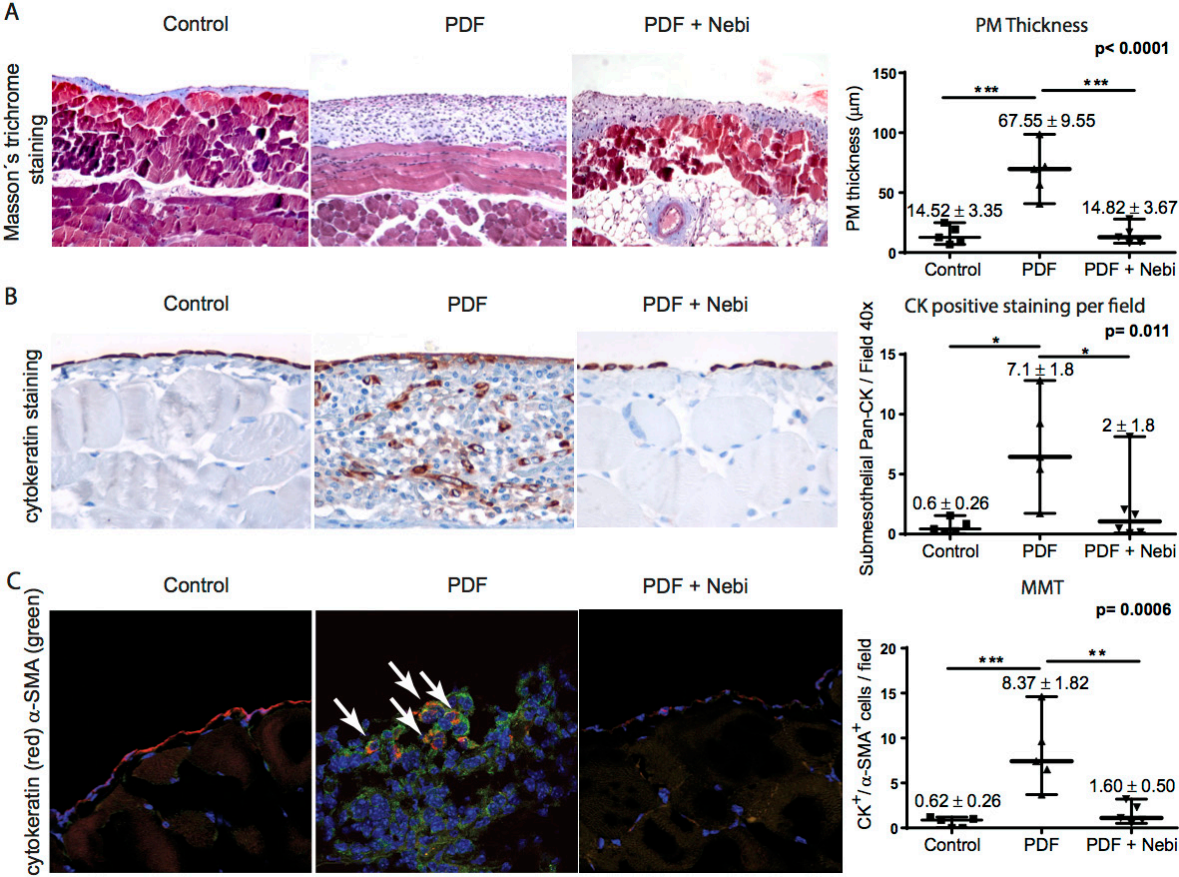
*In vivo* analysis of the peritoneal membrane alterations C57BL/6 female mice were treated or not with PDF or PDF + Nebivolol for 30 days (six mice per group). **A.** Fibrosis assessment by Masson's Trichrome staining. Blue staining indicates collagen depositions. Quantification of peritoneal fibrosis is shown in the corresponding graph. **B.** Immunohistochemistry staining of cytokeratin and quantification of the total cytokeratin positive stained cells in the peritoneal membrane. **C.** Immunofluorescence microscopy analysis of parietal peritoneal tissue sections, stained for cytokeratin (red), and α-smooth muscle actin (α-SMA) (green) and cells nuclei with 4,6-diamidino-2-phenylindole (DAPI) (blue) counterstaining. Number of double positive cells for α-SMA and cytokeratin as per field are shown. Ten randomized calculations per mouse were performed. Data point graphics represent the absolute value of each determination and lines the median, lower and upper range. Numbers on the top of graphics represent the mean ± SE. *P* values < 0.05 are considered statistically significant (one-way Anova test). To account for multiple comparisons, the Bonferroni post-test was used to compare all pairs of means. The symbols represent the statistical differences between the groups (*p < 0.05, **p < 0.01, ***p < 0.001).

We analyzed whether Nebivolol might prevent the PM deterioration in PD mice exposed to PDF. Histological analysis of parietal peritoneal biopsies from PDF group (six in each group) showed a loss of MCs monolayer (cytokeratin positive) and increased PM thickness when compared with control mice group. Interestingly, oral administration of Nebivolol to PD-treated mice (PDF + Nebivolol group) significantly reduced the PM thickness and prevented the loss of MCs monolayer mesothelial (Figure 3A, 3B).

Another histological characteristic of the peritoneum during PD was the accumulation of fibroblasts expressing α-SMA in the submesothelial compact zone; some of them co-expressed cytokeratin (CK), indicating their mesothelial origin (MMT). In PDF group we found an important accumulation of MCs with double positive staining for CK (red) and α-SMA (green) located in the superficial and submesothelial areas (see arrows). In control and PDF + Nebivolol groups, co-stained cells were almost undetectable (Figure 3C).

### Nebivolol decreased the peritoneal angiogenesis and maintained the water and solutes transport capacity

**Figure 4:**
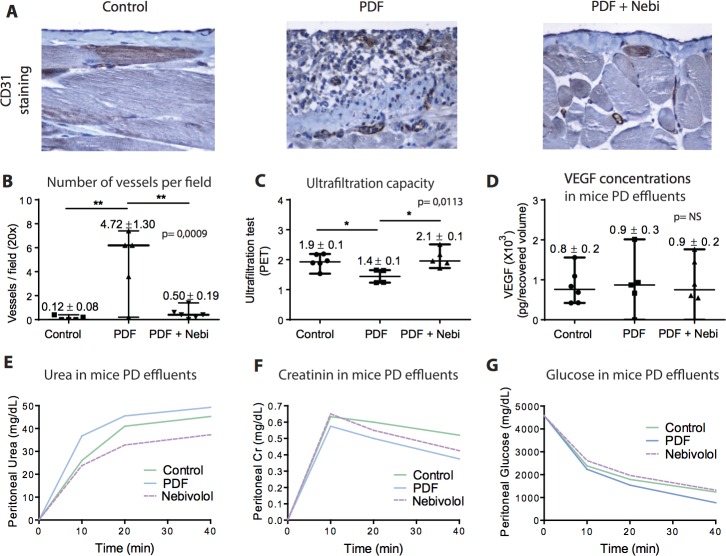
*In vivo* analysis of the alterations related to angiogenesis and the ultrafiltration capacity of the peritoneal membrane **A.** Immunohistochemistry staining of CD31 (vessels) and **B.** quantification of the total CD31 positive stained cells in the peritoneal membrane. **C.** Ultrafiltration capacity analysis (PET test) (30 minutes) after injecting mice with PDF in the last day of the experiment. **D.** Concentrations of VEGF (pg/recovered volume) measured by ELISA in the peritoneal effluents of mice. No significant (NS) differences were observed. **E.**-**G.** Kinetic curves of urea, creatinine and glucose, respectively, in the different groups of mice measured at 10, 20 and 40 minutes. Data point graphics represent the absolute value of each determination and lines the median, lower and upper range. Numbers on the top of graphics represent the mean ± SE. *P* values < 0.05 are considered statistically significant using one-way Anova test, and are depicted in the graphs. To account for multiple comparisons, the Bonferroni post-test was used to compare all pairs of means. NE: MCs with non-epithelioid phenotype. The symbols represent the statistical differences between the groups (*p < 0.05, **p < 0.01, ***p < 0.001).

Angiogenesis is an important component for PM failure and it is associated with alterations in the peritoneal water transport [1]. Recently it has been reported that Nebivolol can regulate the blood vessels formation in cardiovascular system [20]. Herein, we analyzed the effect of Nebivolol on PM angiogenesis and peritoneal water and solute transport. PDF group showed a higher number of CD31^+^ cells in the submesothelial area, indicating an intense neovascularization compared to control and Nebivolol group (Figure 4A, 4B).

In relation to peritoneal transport, after the exposure of the mice peritoneal cavity to 2 ml of PDF during 30 minutes, the PDF group showed an important reduction in UF rate compared to the remaining groups (Figure 4C). However, we did not observe statistical significant differences over time in peritoneal solute transport (urea, creatinine and glucose) among the groups (Figure 4E, 4F, 4G). Likewise, VEGF levels were similar between groups (Figure 4D).

### Nebivolol improved the fibrinolytic capacity in the peritoneal cavity in a PD mice model

**Figure 5:**
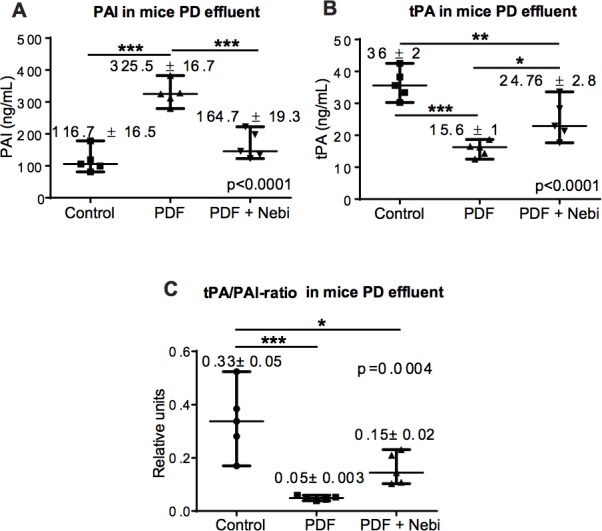
Fibrinolytic capacity *in vivo* **A.**-**B.** Expression of the fibrinolytic factors PAI and tPA in the different mice groups. The levels of these factors were measured in the effluents by ELISA and results are depicted as ng/mL. **C.** The tPA/PAI ratio, an important marker of fibrinolytic capacity, is also shown. Data point graphics represent the absolute value of each determination and lines the median, lower and upper range. Numbers on the top of graphics represent the mean ± SE. *P* values < 0.05 are considered statistically significant using one-way Anova test, and are depicted in the graphs. To account for multiple comparisons, the Bonferroni post-test was used to compare all pairs of means. The symbols represent the statistical differences between the groups (*p < 0.05, **p < 0.01, ***p < 0.001).

Given the improvement of the fibrinolytic capacity found in HOMCs and transdifferentiated HEMCs cultured with Nebivolol or Nebivolol + TGF-β (Figure 2), we measured PAI and tPA concentrations in mice PD effluents after 30 days in PD. PDF exposure was associated to increase PAI (Figure 5A) and decrease tPA levels (Figure 5B), leading to a decrease in fibrinolytic capacity in the peritoneal cavity. In contrast, mice treated with Nebivolol maintained or improved fibrinolytic capacity in the peritoneal cavity.

### Nebivolol partially restored the inflammatory status in the peritoneal cavity from mice treated with PD

Inflammation in peritoneal cavity is one of the most important factors that trigger MMT and PM damage. Bioincompatible PDFs, peritonitis and/or hemoperitoneum are primarily responsible for peritoneal immune activation [2, 3]. Given that Nebivolol seems to have an anti-inflammatory effect on cardiovascular system [21], we decided to measure inflammatory markers in the peritoneal cavity of mice treated with PD. We did not find differences in total peritoneal cells, pro-inflammatory cytokines (IL-6) and anti-inflammatory cytokines (TGF-β and IL-10) between the groups treated or not with Nebivolol. Total cell count, PDF *vs*. PDF + Nebovilol (7.5 ± 0.4 *vs*. 8.8 ± 1.5 × 10^6^ total cell, NS), IL-6 (135 ± 48 *vs*. 30 ± 14 × 10^3^ pg/recovered volume, *p* = 0.07), TGF-β (8785 ± 3810 *vs*. 8297 ± 4295 pg/recovered volume, NS) and IL10 (7.5 ± 4.2 *vs*. 4.6 ± 2.5 × 10^3^ pg/recovered volume, NS), respectively. However, Nebivolol treatment prevented the increase in IL8 values associated to PDF exposure, (674.9 ± 228.6 *vs*. 165.5 62.1 pg/recovered volume, *p* = 0.02).

### Nebivolol did not modify the NO_3_- levels in the peritoneal cavity of mice treated with PD

Nebivolol treatment has generally been associated with increased NO synthesis [22]. NO is a potent vasodilator and stimulates tissue angiogenesis *via* VEGF and HIF dependent pathway [23]. However, we did not find differences in NO_3_- (a marker of NO levels) concentration in mice PD effluent among the groups. PDF *vs*. PDF + Nebovilol groups (175.3 ± 29.8 *vs*. 139.7 ± 45.2 mm/mL, NS, respectively).

## DISCUSSION

PD technique is based on the ultrafiltration capacity and solute clearance of the PM, but its exposure to PDFs leads to PM deterioration. This damage starts with inflammation and MMT of MCs in the PM surface, causing MCs migration, fibrosis, angiogenesis and, consequently, UFF. PD patient's survival frequently depends on maintaining the PM integrity, so its preservation through the employment of more biocompatible PDFs or pharmacological agents, preventing their deleterious effects, is essential for the maintenance of the PD technique [2].

For this purpose and considering that Nebivolol has shown positive cardiovascular effects in non-uremic patients [13], we conduct the present research.

The use of the first β-AR blockers in patients undergoing PD had to be limited due to the possible risk of developing functional and structural PM complications [8, 9]. However there is no clinical experience with the use of new β-AR blockers in PD Patients that contrary seem to have very beneficial systemic effects. The protective effects of Nebivolol on cardiovascular functions have been associated to NO release by endothelial cells, and by its anti-oxidant and anti-fibrotic effects, associated with collagen synthesis reduction and its anti-angiogenic capacity [15]. These protective properties are regulated by the β-AR modulation [16]. Herein, we demonstrate for the first time that peritoneal MCs express β_1_-AR.

Recent data indicate that MCs are very versatile because they express a large number of receptors such as estrogen, PPAR-γ or angiotensin-II [24–26]. Since pleural and peritoneal MCs have the same phylogenetic origin, the discovery of β_2_-AR in pleural MCs is consistent with our results [6] and opens new therapeutic opportunities.

Given the central role of the MMT process in the initiation and progression of peritoneal injury in PD patients [7], [8], [34], we have analyzed the effects of Nebivolol on the MMT of MCs *in vitro* and in a mice PD model. We found that Nebivolol did not inhibit totally the MMT *in vitro* but decreased the ECM synthesis. In addition, it partially prevented the down-regulation of E-cadherin and up-regulation of Snail when MCs were treated with TGF-β (Figure 1).

The loss of fibrinolytic capacity and MCs migration are usually associated with MMT [24]. The following sentence should be: Recently, Shan T. et al. [27] demonstrated that the natural agonist of β-AR, norepinephrine, inducesepithelial-to-mesenchymal transition (EMT) in a gastric adenocarcinoma cell lines, so the blockade of β-AR could inhibit this process.

Herein we demonstrated that Nebivolol treatment increased the tPA-levels in HOMCs and HEMCs supernatant, and effluent of mice in PD. tPA is a natural inhibitor of PAI and its balance maintains an anti-adherent surface. Moreover, elevation of tPA makes a more fluid matrix surface increasing the cell migratory by MMP2 up-regulation, and is also able to inhibit the MMT *via* increases in HGF levels [28]. HGF and BMP7 are natural inhibitors of TGF-β, the master molecule of tissue fibrosis and maximum initiator of the EMT [29].

One would think that the increase in cell migratory ability to submesothelium is a negative phenomenon, but the first requirement to migrate is suffer MMT and tPA blocks MMT via HGF / BMP7 increment [28], therefore tPA increase is always positive.

We demonstrated that Nebivolol also improved the fibrinolytic capacity of MCs mediated by an increase in tPA levels, which is essential to maintain an adequate balance of extracellular matrix components, thereby protecting from the tissue fibrosis and peritoneal adhesions formation. Our results in HEMCs and HOMCs stimulated with TGF-β1 indicate that tPA production, a powerful fibrinolytic factor, decreased together with an increase in PAI, its antagonist. Interestingly, treatments with Nebivolol restored the tPA basal levels or even increased its synthesis above the basal level, decreased the PAI levels and restored the tPA / PAI-ratio. These findings are consistent with the results of Tarighi *et al,* [30] who found an improvement in the fibrinolytic serum profile of hypertensive patients treated with Nebivolol.

Another way by which Nebivolol would inhibit MMT or its deleterious effects is increasing NO [31]. Although we did not observe significant differences in the levels of NO in the mice PD effluent, another possible route by which Nebivolol could inhibit the MMT is increasing the NO synthesis. In mesangial cell culture, a link between NO and regulation of genes related to ECM production has been proposed [32]. NO is able to down-regulate the mRNA and protein levels secretion of modular calcium-binding protein-1 (SMOC-1), and its silencing led to a significant reduction in the expression of TGF-β, PAI-1 and other genes involved in tissue fibrosis and EMT. The downregulation of SMOC-1 was also linked with a disruption in TGF-β signaling by preventing from binding of Smad proteins to the Smad binding element in DNA [31]. In endothelial cells, NO has also been shown to inhibit TGF-β gene expression, through interference with Smad signaling. NO inhibited Smad-2 phosphorylation and nuclear translocation and led to Smad-2 degradation *via* the ubiquitin proteasome pathway. Moreover, it has recently been demonstrated that Nebivolol exerts an anti-inflammatory, anti-fibrotic and anti-migratory effects blocking the same signaling pathway (TGF-β, MMP-2 and MMP9) [21, 33].

Peritoneal inflammation is crucial for the development of PM fibrosis and EMT [1]. Although in this study we observed no statistically significant reduction neither in the pro-inflammatory cytokine IL-6 production nor in the number of total cells in PD-effluent from Nebivolol-treated mice. We did observe differences in IL8 concentrations; this fact and the sum of the sum of pleiotropic effects of Nebivolol may explain its protective effect on PM.

Angiogenesis is another component from PM failure. We found a small number of submesothelial vessels in the group of PD mice treated with Nebivolol. To our knowledge Nebivolol is not considered an anti-angiogenic agent, but it might indirectly attenuate vessels formation through a decrease in tissue fibrosis and MMT. In addition β-AR blockade is associated with anti-angiogenic effects [34, 35].

Since the 80s decade the use of β-blockers in PD patients has been controversial for their association with ultrafiltration rate reduction by decreasing splanchnic blood flow (vasoconstriction) and, possibly, by peritoneal fibrosis induction [8, 9]. However the third generation of β-blockers such as Nebivolol does not produce such effects, in fact Nebivolol increases endothelial NO release inducing vasodilation, anti-oxidation and improving the systemic fibrinolytic capacity [36, 37]. Robust clinical evidences favor that Nebivolol could be approved in US to treat hypertension and in Europe to treat hypertension and heart failure with reduced ejection fraction [37].

Cardiovascular disease remains the leading cause of death in patients on dialysis [38]. The exact causes for this premature vascular aging are complex and include major incidence of hypertension, dyslipidemia, diabetes, oxidative stress, systemic inflammation and tendency to the formation of vascular thrombi with decreased endothelial fibrinolytic capacity. This network has as common factor: the endothelial dysfunction [39], which is associated with deficit of NO by accumulation of a uremic toxin called *N*G monomethyl-L-arginine (L-NMMA) and asymmetric dimethylarginine (ADMA) [40].

Both ADMA and L-NMMA are the result of posttranscriptional methylation of L-arginine residues by protein arginine methyltransferases and are released in their free form following protein hydrolysis. ADMA production is about 10-fold that of L-NMMA and is elevated in patients with chronic renal failure [40]. Plasma levels of ADMA predict adverse cardiovascular events in uremic patients [41], therefore ADMA has been classified as a “uremic toxin” and exhibits adverse cardiovascular effects [39].

Badve SV, et al. published an interesting review and meta-analysis where they analyze the effects of beta-adrenergic antagonists in patients with chronic kidney disease concluding that the beta-blockers treatment in patients with chronic renal failure and chronic systolic heart failure improved all-cause mortality. We believe that in the case of Nebivolol this effect could be due to improvement in the NO synthesis, in the endothelial fibrinolytic capacity and its anti-oxidant and anti-hypertensive effect.

Considering the results presented here and existing pharmacological evidence regarding the cardiovascular protective effects of Nebivolol, we can suggest that this drug may be an excellent candidate for use in PD patients as PM and cardiovascular protector. Future studies with a prospective design, matched and double-blind (if possible) should analyze its therapeutic potential in uremic patients.

## CONCLUSIONS

Nebivolol displays a protective effect against PM damage induced by PD fluids. This protection involves a partial anti-MMT, anti-fibrotic, anti-angiogenic and pro-fibrinolytic effects. Importantly, the protective cardiovascular effects of this drug make it a very attractive candidate to be used in PD patients.

## MATERIALS AND METHODS

### Isolation, culture and treatment of HOMCs and HEMCs

HOMCs were obtained from omental samples taken from patients undergoing elective abdominal surgery and from effluents of PD patients as described previously [42–45].

HEMCs were isolated from a PD overnight exchange of patients with more than 3 months in PD as previously described by us [46].

The purity of the omentum- and effluent-derived MCs cultures was determined by the expression of the standard mesothelial markers: intercellular adhesion molecule (ICAM)-1, calretinin and cytokeratins. These MCs cultures were negative for von-Willebrand factor and CD45, ruling out any contamination by endothelial cells or macrophages [43–45].

To induce MMT *ex vivo*, HOMCs were seeded on wells coated with collagen I (50 mg/ml, Roche Diagnostics GmbH) and treated for 48 h with human-recombinant TGF-β1 (1 ng/ml, R&D Systems), a commonly used *in vitro* model of MMT [26, 43–45, 47].

To evaluate the effect of β-AR blockade on MMT prevention, HOMCs were co-treated with TGF-β and Nebivolol (Menarini, Italy) at of 10 and 15 nM during 6, 48 and 72 hours, as referred by others [48]. We also analyzed the effect of Nebivolol on spontaneously transdiffferentiated mesothelial cells isolated from peritoneal effluent of PD patients; HEMCs were also treated with Nebivolol (10 and 15 nM) and analyzed at 48 hours. These cells were characterized by non-epitheliod morphology, E-cadherin down-regulation and Snail and mesenchymal markers up-regulated.

The present study adjusts to the Declaration of Helsinki and was approved by the Ethics Committee of the Hospital Universitario de La Princesa, Madrid, Spain. Informed written consent was obtained from all the patients (PD effluent and omentum donors).

### Western blot analysis and quantitative RT-PCR

For western blotting, MCs cultures were lysed in a buffer containing 1% sodium deoxycholate and 0.1% sodium dodecyl sulfate (SDS). The total protein was quantified using a protein assay kit (Bio-Rad). Total cell protein (50 mg) was resolved on 8-10% SDS-polyacrylamide gels and transferred onto nitrocellulose membranes, which were then blocked with fat-free milk and probed with specific antibodies against b1 adrenergic receptor (Santa Cruz Biotechnology). Images of the blots were acquired with a LAS-1000 Charge Coupled Device camera (Fujifilm).

For quantitative RT-PCR analysis, MCs were lysed in TRI Reagent (Ambion), and RNA was extracted according to manufacturer's instructions. Complementary-DNA was synthesized from 2 μg of total RNA by reverse transcription (RNA PCR Core Kit, Applied Biosystems). Quantitative PCR was carried out in a Light Cycler 2.0 using a SYBR Green Kit (Roche Diagnostics GmbH) and specific primers sets for human Snail, E-cadherin, fibronectin, pro-collagen-I, α-SMA and histone H3. Samples were normalized with respect to the value obtained for H3. All experiments were repeated at least three times. For RT-PCR determinations in each experiment it was assigned a value of one to a control omentum (C-) from which the relative values of the remaining groups were calculated. The primer sets used are depicted in Table S1.

### Fibrinolytic capacity and enzyme-linked immunoassays in HOMCs and HEMCs

To evaluate the effect of Nebivolol on fibrinolytic capacity, HOMCs and HEMCs were cultured with TGF-β Nebivolol 10 and 15 nM during 48 hours. The plasminogen activator inhibitor type-1 (PAI-1) and tissue-type plasminogen activator (tPA) concentration were measured in culture supernatants by ELISA kits (PAI-1, BioVentor Laboratorni Medicina, a.s. Karasek, Czech Republic) and (R&D Systems Inc; Minneapolis, USA), respectively.

Using the same technique we also measure VEGF-A, TGF-β and IL-6 in MCs culture supernatants (VEGF-A and TGF-β; R&D Systems Inc; Minneapolis, USA and IL-6: BD Biosciences Pharmingen, San Diego, CA).

### Peritoneal dialysis fluid exposure model in mice

This study was performed in 21 non-uremic female C57BL/6 mice (*N* = 18, aged 12-14 weeks; Harlan Interfauna Iberica, Spain). The experimental protocol was approved by the Animal Ethics Committee of the Unidad de Cirurgía Experimental of Hospital La Paz, Madrid, Spain. The animals had free access to food and water. A customized peritoneal catheter (Access Technologies) was surgically placed in all mice as previously described by González-Mateo, *et al* [17]. After one week, animals were divided into three groups: 6 controls (exposed to the catheter alone), 6 animals to receive daily infusions of 2 ml of 4,25% glucose PDF (*Stay Safe*, Fresenius Medical Care) and 6 animals to receive 2 ml PDF peritoneal infusions and Nebivolol diluted in water (8 mg/kg/day in 15 μL of volume) orally. The dropout animals were mainly due to catheter displacement (one from each group).

### Histological analysis

Peritoneum fragments were fixed in Bouin's fixative for 24 h and then embedded in paraffin. 5 μm sections were stained with Masson's Trichrome for submesothelial thickness determination. Thickness was measured microscopically every 60 mm for the whole length of the biopsy (Leica CTR6000 with LAS-AF6000; Leica Microsystems).

For immunofluorescence stainings, tissue was embedded in OCT (Optimal Cutting Temperature) medium and cut into 5 mm sections. The frozen sections were fixed for 15 minutes in 4% formaldehyde in PBS and blocked with 10% horse serum for 1 hour in PBS with 0.3% Triton X-100. Then samples were stained with primary antibodies anti-pan cytokeratin (CK), α-SMA (Sigma-Aldrich, Saint Louis, Missouri, USA) and CD31 (Beckton Dickinson). Afterwards, they were conjugated with zenon compounds (Invitrogen). Nuclei were stained with DAPI. Micrography was performed with a fluorescence microscope (Leica CTR6000 with LAS-AF6000).

For immunohistochemistry stainings, biopsies were fixed in paraformaldehyde, embedded in paraffin and cut into 3 μm sections. After paraffin remotion with xylol treatment, samples were heated to expose any masked antigens using a Real Target Retrieval Solution containing citrate buffer (pH 6.0, Dako). Samples were pre-treated with Real Peroxidase-Blocking Solution (Dako) to block the endogenous peroxidase. Tissue sections were stained with anti-pan cytokeratin or anti-CD31 and counterstained with nuclear hematoxylin. Images were analyzed by computerized digital image analysis (AnalySIS, Soft Imaging System). Positive staining was counted and expressed as the mean of 10 independent counts for each animal, quantified at 20x using the analysis program Image-J 1.37c (National Institute of Health, USA).

### Peritoneal function assessment

After the 30-days treatment, 2 ml of PDF were instilled into the peritoneal cavity and after 10, 20 and 40 minutes, 300 μl of effluent were obtained through direct puncture of the abdominal wall for urea, Creatinine and glucose dosing (Servicio de Bioquímica Clínica from Laboratorio de Bioquímica from Hospital Universitario La Paz, Madrid, Spain). On next day, 2 ml of PDF were infused into the peritoneal cavity and after 30 minutes animals were anesthetized by halothane inhalation and sacrificed by cervical dislocation. Total peritoneal effluent volume was collected and weighed on a precision balance for the evaluation of ultrafiltration capacity. Afterwards, effluents were centrifuged and total cells were counted. Effluents were stored at −80ºC to later analyze different parameters.

### Evaluation of the fibrinolytic capacity in of HOMCs, HEMCs and mice mouse PD effluent

To evaluate the fibrinolytic capacity of human MCs (HOMCs and HEMCs), these cells were co-cultured with TGF-β1 and Nebivolol 10 and 15 nM during another 48 h Cell culture supernatants were collected and stored to −80ºC. Concentrations of plasminogen activator inhibitor type-1 (PAI-1, BioVentor Laboratorni Medicina, a.s. Karasek, Czech Republic) and of tissue-type plasminogen activator (tPA, R&D Systems) were measured using ELISA kit following manufacturer's instructions. The values in supernatant are expressed in ng/mg protein. Mice PD effluent samples were collected after 30 days of PD. In PD mice effluent, PAI and tPA were also determined using the same Elisa Kits. The values are expressed in ng/mL (UF volume).

### Enzyme-linked immunoassays (ELISA) and nitrates (NO3-) in MCs cultures and mice PD effluents

TGF-β1, VEGF and IL-6 protein levels were measured in human MCs supernatants by ELISA according to manufacturer's instructions. In mice, PD effluent samples were collected after 30 days in PD. VEGF-A, TGF-β_1_, IL-6, IL-8 and IL-10 were measured by ELISA, (VEGF-A and IL-8: Bender Med Systems, Vienna, Austria; TGF-β_1_: R&D Systems, Minneapolis, USA; and IL-6 and IL-10: BD Biosciences Pharmingen, San Diego, CA). NO_3_- was also determined in MCs supernatant and PD effluent by capillary electrophoresis as described [49]. The values are expressed in pg/total recovered volume.

### Statistical analysis

In Figure 1, the results (qPCR) are presented as mean ± SE in bars graphics. In Figures 2 to 5 the results are showed in data point graphics representing the absolute value of each determination and lines the median, lower and upper range. Numbers on the top of graphics represent the mean ± SE. The difference between the two groups was calculated using the nonparametric Mann-Whitney rank sum. The global difference between tree groups was calculated using ANOVA one way and Bonferroni post-hoc test. We used the SPSS statistic package version 14.5 (Chicago, IL) and GraphPad Prism version 5.0 (La Jolla, CA). *P* < 0.05 was considered statistically significant.

## SUPPLEMENTARY TABLES


