# Correction: Nebivolol, a β_1_-adrenergic blocker, protects from peritoneal membrane damage induced during peritoneal dialysis

**DOI:** 10.18632/oncotarget.16782

**Published:** 2017-04-03

**Authors:** Georgios Liappas, Guadalupe González-Mateo, Anna Rita Aguirre, Hugo Abensur, Patricia Albar-Vizcaino, Emilio González Parra, Pilar Sandoval, Laura García Ramírez, Gloria del Peso, Juan Manuel Acedo, María A. Bajo, Rafael Selgas, José A. Sánchez Tomero, Manuel López-Cabrera, Abelardo Aguilera

**Present**: The currently listed funding information is incorrect.

**Corrected**: The proper funding information is listed below.

Original article: Oncotarget. 2016; 7(21):30133-30146. doi: 10.18632/oncotarget.8780

**FUNDING**

This work was supported by Menarini Spain, grants PI 15/00598 from Fondo de Investigaciones Sanitarias (FIS) Carlos III Institute to A.A.; SAF2013-47611R from The Ministry of Economy and Competitiveness and grant S2010 / BMD-2321 (FIBROTEAM Consortium) from Autonomous Community of Madrid to M.L.-C .; G.L. Was Fully supported from the European EuTRiPD Marie Curie Project.

