# Supplementary material for: Nebivolol, a β1-adrenergic blocker, protects from peritoneal membrane damage induced during peritoneal dialysis
Source: Oncotarget. 2016 Apr 18;7(21):30133–46. doi: 10.18632/oncotarget.8780 (PMC5058669; doi:10.18632/oncotarget.8780)
Supplement: Supplementary file 1 [file oncotarget-07-30133-s001.pdf]

Nebivolol, a β_1-adrenergic blocker, protects from peritoneal membrane damage induced during peritoneal dialysis

Supplementary Material

Table S1: Specific human primers for quantitative RT-PCR

**Gene**	**Forward primer**	**Reverse primer**
E-Cadherin	5´TAAGGTGACAGAGCCTCTG3´	5´TGGGTGAATTCGGGCTTGTT3´
Fibronectin	5´CCTGAAGCTGAAGAGACTTGC3´	5´CGTTTCTCCGACCACATAGGA3´
Pro-collagen	5´GCTATGATGAGAAATCAACCG3´	5´GCTTCCCCATCATCTCCATTC3´
α-SMA	5′CCGACCGAATGCAGAAGGA3′	5′ACAGAGTATTTGCGCTCCGAA3′
Snail	5’GCAGAAGGAGGAGGGCAGAAT3’	5’TATGTGCTGGCCTTGGTGAGG3’
Histone H3	5´AAAGCCGCTCGCAAGAGTGCG3´	5´ACTTGCCTCCTGCAAAGCAC3´
